# Misexpression of *Pknox2* in Mouse Limb Bud Mesenchyme Perturbs Zeugopod Development and Deltoid Crest Formation

**DOI:** 10.1371/journal.pone.0064237

**Published:** 2013-05-22

**Authors:** Wenrong Zhou, Huang Zhu, Jianzhi Zhao, Hanjun Li, Yong Wan, Jingjing Cao, Haixia Zhao, Jian Yu, Rujiang Zhou, Yiyun Yao, Lingling Zhang, Lifang Wang, Lin He, Gang Ma, Zhengju Yao, Xizhi Guo

**Affiliations:** Bio-X Institutes, Key Laboratory for the Genetics of Developmental and Neuropsychiatric Disorders (Ministry of Education), Shanghai Jiao Tong University, Shanghai, China; Cincinnati Children's Hospital Medical Center, United States of America

## Abstract

The TALE (Three Amino acid Loop Extension) family consisting of Meis, Pbx and Pknox proteins is a group of transcriptional co-factors with atypical homeodomains that play pivotal roles in limb development. Compared to the in-depth investigations of Meis and Pbx protein functions, the role of Pknox2 in limb development remains unclear. Here, we showed that *Pknox2* was mainly expressed in the zeugopod domain of the murine limb at E10.5 and E11.5. Misexpression of *Pknox2* in the limb bud mesenchyme of transgenic mice led to deformities in the zeugopod and forelimb stylopod deltoid crest, but left the autopod and other stylopod skeletons largely intact. These malformations in zeugopod skeletons were recapitulated in mice overexpressing *Pknox2* in osteochondroprogenitor cells. Molecular and cellular analyses indicated that the misexpression of *Pknox2* in limb bud mesenchyme perturbed the *Hox10-11* gene expression profiles, decreased *Col2* expression and Bmp/Smad signaling activity in the limb. These results indicated that *Pknox2* misexpression affected mesenchymal condensation and early chondrogenic differentiation in the zeugopod skeletons of transgenic embryos, suggesting *Pknox2* as a potential regulator of zeugopod and deltoid crest formation.

## Introduction

The vertebrate limb bud arises from the lateral plate mesoderm and then develops into three segments (including the stylopod, zeugopod and autopod) along the proximal-distal (PD) axis through an endochondral ossification mechanism. Limb patterning and morphogenesis are established by diffusible signals originating from different domains of the limb bud, including the Fgf, Wnt and RA signaling pathways [1]. In concert with these instructive morphogens, *Hox* genes (paralogous groups 9–13) are expressed in restricted domains along the axes of the limb buds in spatial and temporal colinearity, and provide positional information for limb patterning, skeletal condensation and differentiation. Expression of the *Hoxd 9-13* genes in the limb bud is activated sequentially from *Hoxd9* to *Hoxd13* at the posterior border of the limb bud. The *Hoxa9-13* genes are activated similarly to the *Hoxd9-13* genes. The sequential activation of these genes correlate with the malformations in the limb skeletons of specific *Hox* mutants. For instance, compound mutants of *Hoxa9/d9* have a shorter humerus and a loss of deltoid crest formation in the forelimb stylopod [2]. Quadruple *Hox9* (*Hox9aabbccdd*) mutant mice exhibit severe forelimb defects with a loss of posterior skeletal elements including complete autopod loss and partial zeugopod loss, suggesting a role of *Hox9* in forelimb anterior-posterior patterning [3]. The triple inactivation of *Hox10* (*Hox10aaccdd*) results in severe agenesis in the hindlimb styplopod, and defects in the forelimb zeugopod and deltoid crest to a lesser degree [4], [5]. Double mutants of *Hoxa11/d11* selectively display deformities in the forelimb zeugopod [4], [6], and triple mutants (*Hox11aaccdd*) demonstrate dramatic malformations of the fore- and hindlimb [5]. Additionally, double mutants of *Hoxa13/d13* exhibit malformations in autopod development [7]. Elimination of all *Hoxa* and *Hoxd* genes results in early arrest of limb outgrowth, with severe truncations in distal elements [8]. These functional analyses highlight the synergistic and redundant role of the *Hox* genes in limb development: *Hox9* participates in stylopod specification, *Hox10/11* contributes to zeugopod formation and *Hox12/13* regulates autopod development.

While the *Hox* genes have a universal role in a variety of developmental processes, the specificities of *Hox* function are achieved by interactions with co-factors such as the members of the TALE (Three Amino acid Loop Extension) superfamily. The TALE superfamily consists of transcription factors with atypical homeodomains including the Pbx, Meis and Pknox proteins. Similar to the *Hox* genes, these *TALE* genes are dynamically expressed in the limb bud. Genetic studies reveal that *TALE* genes regulate limb patterning and development of the skeletal elements. For instance, *Pbx1* is exclusively expressed in the proximal limb bud, whereas *Pbx2* is expressed throughout the limb mesenchyme [9]. *Pbx1*-deficient mice have malformations in the proximal limb elements, while double *Pbx1/2* mutants exhibit distal limb deformities in addition to the proximal limb defects [9], [10]. *Meis1* is a specific marker for the proximal domain, especially for the presumptive stylopod skeleton in early limb bud [11]. Overexpression of the *Meis1* gene in the limb bud shifts limb PD patterning and promotes the formation of proximal limb segments [11], [12]. The TALE transcriptional factors could exert their effect by forming a heterotrimeric complex, such as Pbx-Meis-Hox or Pknox-Pbx-Hox, adding DNA binding specificity and affinity to the Hox proteins [10], [13]. In addition, Pbx-Hox complex could directly activate the *Shh* expression as well as *Hox* expression in the regulation of limb development [9]. The Pknox subfamily members *Pknox1/2* are also dynamically expressed in the avian limb bud [14], [15]. *Pknox1*-null mutation is lethal in mice at E7.5 and hypomorphic *Pknox1* mutant mice exhibit no obvious alterations in limb development [16], [17]. However, the role of *Pknox2* protein in limb development remains unclear.

Here, we overexpressed the *Pknox2* genes in limb mesenchyme or osteochondroprogenitor cells in the early limb bud in transgenic mice. Misexpression of *Pknox2* in the limb bud mesenchyme resulted in deformities in zeugopod elements and a loss of deltoid crest formation. The malformations in these transgenic mice were correlated with the perturbations of *Hoxd10-11* gene expression profiles in the zeugopod elements. Therefore, *Hox*-dependent patterning alterations underlie, at least in part, the limb zeugopod defect in mice of *Pknox2* misexpression.

## Materials and Methods

### Generation of Transgenic Mice

The 1.4 kb coding sequence CDS of *Pknox2* gene (BC050865, ATCC) was cloned into a vector harboring the *Prx1* promoter [18], *Col2a1* promoter [19] or rat *Col1a1-*3.6 kb promoter [20], as previously described. Transgenic embryos were generated by microinjecting the linearized construct into fertilized oocytes of the ICR strain. Transgenic mouse strains were descendants from the founder animals. For genotyping, genomic DNA was extracted from mouse tails. Forward oligo used for *Prx1-Pknox2* transgenic mice: 5′- TCTGGTGGCAGCGAAAGTC-3′; forward oligo for *Col2a1-Pknox2* transgenic mice: 5′-AGGGTGTTGTTTAGAATGGGA-3′; forward oligo for *Col1a1-Pknox2* transgenic mice: 5′-CACTCCAGTGACAGCACCTCT-3′; reverse oligo for transgenic mice: 5′-ATGGAGGATAGTTCAGGGCTT-3′.

### Ethics Statement

All the mouse embryonic manipulations comply with the guidelines of the Bioethics Committee of Bio-X Institutes of Shanghai Jiao Tong University (SYSK-SH-2011–0112).

### Skeletal Preparations

Mouse embryos at P0 were eviscerated, and their skins were removed. Mice were fixed overnight in 95% ethanol followed by staining overnight in Alcian Blue and Alizarin Red solution (SIGMA) as previously reported [21].

### Histology, *in situ* Hybridization and Immunohistochemistry

For histology and *in situ* hybridization, embryos were sacrificed at various ages, dissected, and fixed in 4% paraformaldehyde (PFA)/PBS at 4°C overnight. After fixation, the tissues were dehydrated in 100% ethanol and embedded in paraffin. The embedded tissues were cut to generate 8 µm-thick sections and mounted onto slides. HE staining and Safranin O staining were performed following standard protocols. Whole mount *in situ* hybridization were performed as described [22]. Immunohistochemistry was conducted with antibody MyoD (BD, Cat#554130) and Col1a2 (Millipore, Cat#AB765P).

## Results

### *Pknox2* is Mainly Expressed at the Zeugopod and Partial Stylopod Domains During Limb Bud Outgrowth

**Figure 1:**
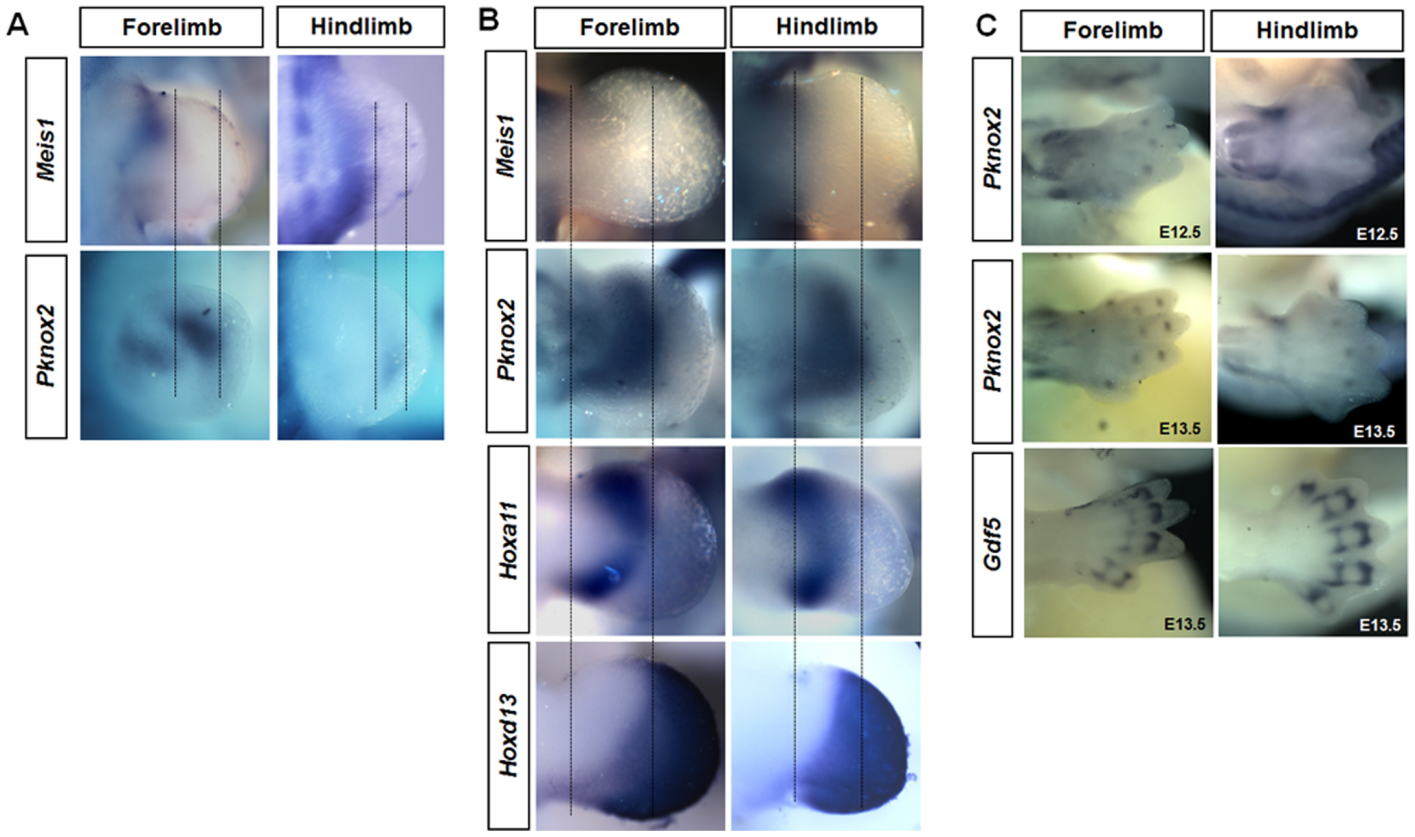
*Pknox2* is expressed in the zeugopod and partial stylopod domains during limb bud outgrowth. **A:** **** Comparative expression of *Meis1* and *Pknox2* in limb bud at E10.5. **B:** Comparative expression of *Meis1, Pknox2, Hoxa11* and *Hoxd13* in limb bud at E11.5. *Pknox2* is mainly detected in the zeugopod domain of both the forelimb and hindlimb at E10.5 and E11.5 and has a similar and partially overlapping pattern with *Hoxa11*. Meanwhile, *Pknox2* expression is partially present in the stylopod domain of the forelimb at E10.5 and E11.5. The domains between two vertical guidelines are presumptive zeugopod. **C:** *Pknox2* expression exists in the zeugopod domains and joint regions of the autopod at E12.5 and E13.5.

*Pknox2* expression has been detected throughout the limb mesenchyme and is particularly strong in the mesenchyme underlying the ectoderm in the chick limb bud [15]. We examined *Pknox2* expression during mouse limb bud development. *Meis1*, *Hoxa11* and *Hoxd13* are used as markers for the stylopod, zeugopod and autopod, respectively [12], [23]. Compared with the expression of those markers, *Pknox2* expression was first detected as a stripe in the central region of the hindlimb at E10.5, which was between the *Meis1*-expression domain and the progress zone (right panel of Fig. 1A). At E11.5, *Pknox2* expression mostly overlapped with the *Hoxa11*-expression domain in the zeugopod, distinct from the *Meis1* and *Hoxd13*-expression domains. Of note, the expression of *Pknox2* was strong in the intermediate region, whereas *Hoxa11* was preferentially expressed in the anterior and posterior borders of the presumptive zeugopod (Fig. 1B). In addition, *Pknox2* was not only detected in similar regions in the forelimb as that in the hindlimb at E10.5 and E11.5, but was also partially present in the stylopod domain of the forelimb (left panel of Fig. 1A, B). At stages E12.5 and E13.5, *Pknox2* expression was also detected in the digit joint region in the autopod in addition to the zeugopod domain (Fig. 1C), which was indicated by *Gdf5* expression. Taken together, *Pknox2* expression was mainly restricted to the zeugopod domains of both the forelimb and hindlimb buds. It was also partially expressed in the stylopod domain of the forelimb as well as the digit joint regions.

### Ectopic Expression of *Pknox2* in the Limb Mesenchyme Progenitors Impairs Zeugopod Development

**Figure 2:**
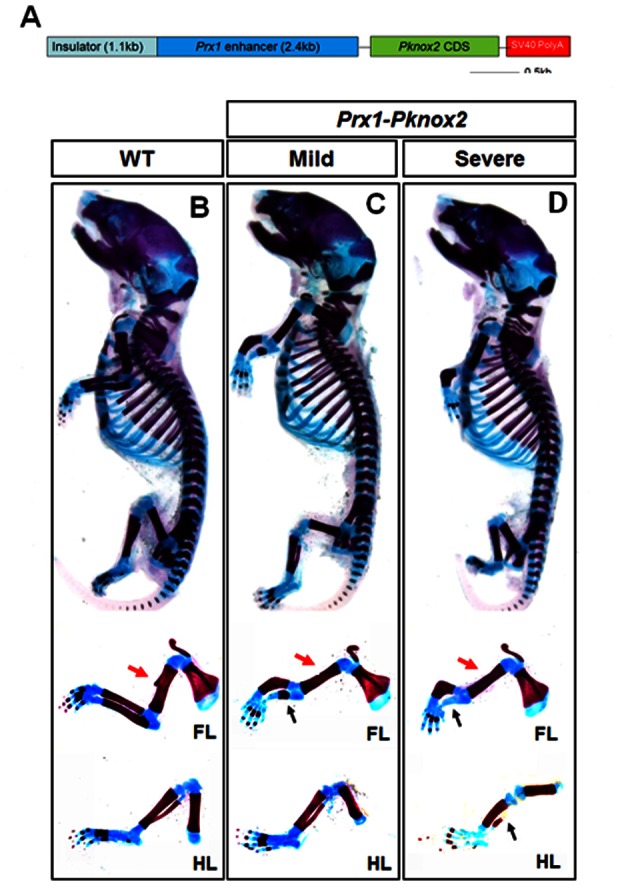
Skeletal preparations for *Prx1-Pknox2* transgenic mice. **A:** Schematic diagram for *Prx1-Pknox2* construct. **B:** WT embryo. **C-D:** *Prx1-Pkonx2* transgenic mice with mild (C) and severe phenotypes (D). Transgenic mice display a shortened radius and ulna with impaired ossification (arrows, n = 3). The deformity in the forelimb zeugopod is much more severe than in the hindlimb (n = 3). All samples are collected at P0. FL, forelimb; HL, hindlimb.

To investigate the role of *Pknox2* in limb development, we overexpressed *Pknox2* cDNA in the mouse limb mesenchyme under the control of the 2.4 kb promoter of *Prx1* gene [18], represented by the diagram in Figure 2A. The *Prx1* promoter ectopically drove *Pknox2* expression in the whole limb bud mesenchyme from E9.5 onward. Three stable transgenic lines were obtained with different phenotypic severities. According to the skeletal preparations at P0, they displayed obvious defects in the zeugopod elements of the forelimb and hindlimb, including a bent anterior radius/tibia and shortened ulna/fibia with disruption of ossification (black arrows in Fig. 2B–D). Yet the deformities were less severe in the hind limb compared to the forelimb (Fig. 2D). Additionally, the deltoid crest of forelimb stylopod was missing in the transgenic mice (red arrows in Fig. 2B–D). However, the autopod and other stylopod skeletons were largely intact in the transgenic mice. Moreover, the sternum, axial, facial and cranial skeletons were relatively normal in the *Prx1-Pknox2* mice, even though *Prx1* promoter has various activity in these tissues. Our findings suggest that *Pknox2* misexpression selectively affects limb zeugopod development.

**Figure 3:**
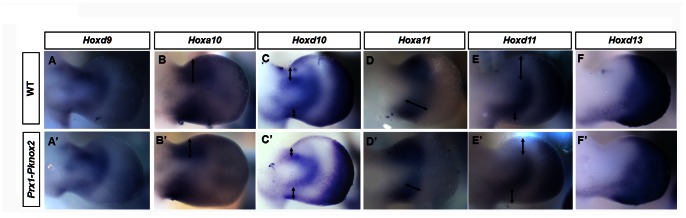
Alterations in *Hox* gene expression in the limb of *Prx1-Pknox2* embryos. **A-F’:** Expression of *Hoxd9* (A, A’), *Hoxa10* (B, B’), *Hoxd10* (C, C’), *Hoxa11* (D, D’), *Hoxd11* (E, E’) and *Hoxd13* (F, F’) in WT (A-F) and *Prx1-Pknox2* embryos (A’-F’) in E11.5 forelimbs. The *Hoxa10*, *Hoxd10* and *Hoxd11* expression domains are anteriorly shifted (double arrows in B-C’, E, E’), whereas the *Hoxa11* expression domain is shortened in the central zeugopod region (double arrows in D, D’) (n = 3). The limb is oriented so that the anterior is on the top and the posterior is on the bottom.

To molecularly characterize the zeugopod defects in the *Prx1-Pknox2* transgenic mice, we examined the expression of zeugopod-related *Hox* genes in the forelimb at E11.5 by whole mount *in situ* hybridization. By E11.5, limb skeletal progenitor cells begin to condense and undergo chondrogenic differentiation. Compared to wild type (WT) controls, the expression domains of *Hoxa10*, *Hoxd10* and *Hoxd11* in *Prx1-Pknox2* transgenic mice were anteriorly shifted (arrows in Fig. 3B, B’, C, C’, E and E’) and the posterior region of the *Hoxa11*-expression domain was shortened (arrows in Fig. 3D, D’) (n = 3/3). In contrast, the expression of *Hoxd9* and *Hoxd13* were largely unchanged (Fig. 3A, A’, F and F’) (n = 3/3). In addition, the expression levels of these *Hox* genes were not significantly altered based on qRT-PCR (Fig. S1). Therefore, the abnormality of zeugopod elements in the *Prx1-Pknox2* transgenic mice might be correlated with alteration of the expression domains of zeugopod-related *Hox* genes.

### Ectopic Expression of *Pknox2* in Osteochondroprogenitor Cells Recapitulates the Forelimb Defects

**Figure 4:**
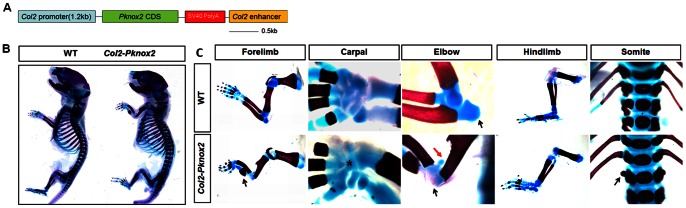
Skeletal preparations of *Col2-Pknox2* transgenic mice. **A:** Schematic diagram for *Col2-Pknox2* construct. **B:** Whole skeletons of WT and *Col2-Pknox2* transgenic mice at P0. **C:** High-power view of the forelimb, carpal bones, elbow and hindlimb at P0. In comparison to WT, *Col2-Pknox2* transgenic embryos exhibit defects including a bent radius and ulna with impaired ossification (black arrows), partially fused carpal bones (star) in the wrist, deformed elbow (black arrow: missed olecranon; red arrow: ectopic cartilage) and ectopic rib formation at lumbar vertebrae (L1, black arrow). N = 3.

To examine whether *Pknox2* itself had a preferential activity in the skeletal progenitors, we generated *Col2-Pknox2* transgenic mice that ectopically expressed *Pknox2* in chondroprogenitor cells and most of the chondrocytes in all the skeletons (Fig. 4A). Three stable *Col2-Pknox2* transgenic lines were generated and their skeletal preparations were analyzed at P0 (Fig. 4B). Compared with the *Prx1-Pknox2* transgenic mice, similar but less severe phenotypes were observed in the *Col2-Pknox2* mice, including partially fused carpals, anteriorly bent and shorter radius, and delayed ossification in forelimb zeugopod elements (Fig. 4C). The olecranon was missing and ectopic cartilage was formed in the upper side of the elbow joint region in *Col2-Pknox2* mice (arrows in Fig. 4C). In addition, a small ectopic rib-like cartilage structure was detected in the L1 vertebrae of mutant axial skeletons; this structure was not observed in the control embryos (Fig. 4C). This result suggests that a partial homeotic transformation occurred in the axial skeletons of *Col2-Pknox2* mice. In contrast, deltoid crest formation was lost in *Prx1-Pknox2* mice but was relatively normal in *Col2-Pknox2* mice. Interestingly, we found that the hindlimb skeletons in *Col2-Pknox2* mice were also largely intact, suggesting that misexpression of *Pknox2* selectively disrupts the forelimb zeugopod development.

**Figure 5:**
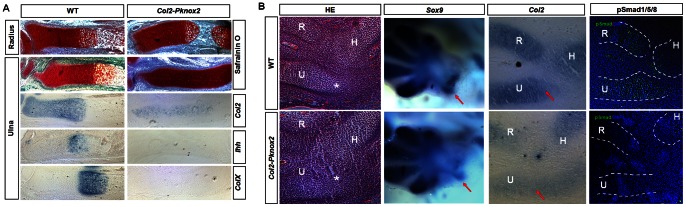
Disruption of chondrocyte differentiation and ossification in the zeugopod elements of *Col2-Pknox2* mice. **A:** Chondrocyte differentiation is blocked at an early stage in *Col2-Pknox2* transgenic embryos compared to WT, as revealed by Safranin O staining and *in situ* hybridization of *Col2*, *Ihh* and *ColX* in the ulna at E15.5 (50X, n = 2). **B:** HE staining showed that the outline of ulna and radius skeleton in the transgenic embryo was not as clear as that of WT (left panel). The expression levels of *Col2* and p-Smad1/5/8, but not *Sox9*, were down-regulated in the ulna and radius skeletons of transgenic mice compared to WT controls at E12.5 (100X, n = 3), indicating that the chondrocyte condensation and differentiation is impaired upon *Pknox2* overexpression.

To gain insight into the timing and appearance of the defects in *Col2-Pknox2* mice, we analyzed forelimb zeugopod development by *in situ* hybridization and immunohistochemistry (IHC). Safranin O staining on the sections at E15.5 showed that chondrocyte hypertrophy and bone ossification were delayed in the radius and ulna (Fig. 5A). The chondrogenic differentiation was severely suppressed in the ulnas from *Col2-Pknox2* mice at E15.5, as indicated by the decreased expression of *Col2*, *Ihh* and *ColX* (Fig. 5A), which are markers for non-hypertrophic, prehypertrophic and hypertrophic chondrocytes, respectively. At E12.5, HE staining showed that no clear condensation of the olecranon was observed and that the cells in this region were loosely organized in the transgenic embryos compared to WT controls (stars in Fig. 5B). In fact, early condensation and chondrogenic differentiation in the zeugopod elements were impaired, as revealed by the decreased *Sox9* and *Col2* expression in the proximal elbow-joint region of the transgenic mice at E12.5 (arrows in Fig. 5B). We also examined zeugopod-related *Hox* gene expression patterns in the E11.5 *Col2-Pknox2* transgenic embryos, but no visible changes were detected (data not shown). Collectively, these results suggest that misexpression of *Pknox2* disrupts zeugopod formation at the condensation stage and during early chondrogenic differentiation.

Bmp signaling is an important regulator of chondrogenesis during endochondral ossification. *Bmp7* and *BmpR1b* double knockout mice have a nearly absent ulna and a shortened and bent radius [25]. To test whether overexpression of *Pknox2* affects Bmp signaling activity, the protein expression of p-Smad1/5/8 was examined in the ulna and radius of *Col2-Pknox2* transgenic embryos at E12.5 via IHC. Interestingly, p-Smad1/5/8 expression levels were markedly decreased in the developing ulna and radius of transgenic mice, whereas p-Smad1/5/8 expression levels were relatively normal in the humerus of transgenic mice and WT controls (Fig. 5B). These findings indicate that the effect of *Pknox2* misexpression on zeugopod condensation and differentiation is partially mediated by the Bmp signaling pathway.

### Ectopic Expression of *Pknox2* in Osteoblasts Blocks Deltoid Crest Formation in the Forelimb

**Figure 6:**
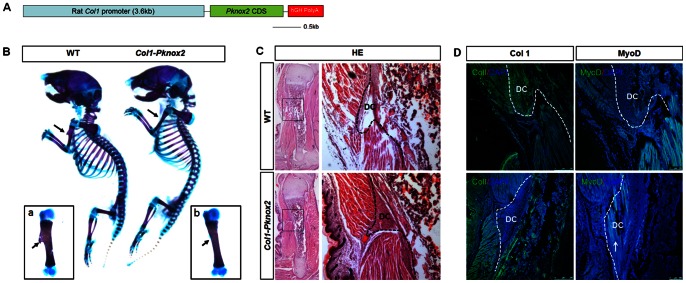
*Col1-Pknox2* transgenic mice lack deltoid crest in the forelimb. **A:** Schematic diagram for *Col1-Pknox2* construct. **B:** Skeletal preparations for *Col1-Pknox2* transgenic mice at P0. A missing deltoid crest is observed in transgenic mice (arrows in b) compared to WT (a). **C:** HE staining of the deltoid crest (DC) in the forelimb. The right panel is a high-power view of the boxed regions in the left panel (50X, n = 3). **D:** IHC analyses for deltoid crest formation in the forelimb. IHC with antibodies against Col1 and MyoD revealed that elongated muscle cells (white arrow in lower panel) existed at the presumptive deltoid crest (DC) of the *Col1-Pknox2* transgenic mice, which was not detected in the WT controls (upper panel). Dashed lines outline the deltoid crest tissues (50X, n = 3). DAPI was used to mark cell nuclei.

To investigate whether *Pknox2* regulates zeugopod development at later stages of osteoblast differentiation, we also ectopically overexpressed *Pknox2* in osteoblast lineages by generating *Col1-Pknox2* transgenic mice (Fig. 6A). In these transgenic mice, *Pknox2* expression was driven by the 3.6 kb rat *Collagen I* promoter (Col1a1 3.6), which was active as early as the osteoblast progenitor stage. Interestingly, there were no apparent defects in other skeletal elements except for the missing deltoid crest in the forelimbs of the *Col1-Pknox2* transgenic mice (arrows in Fig. 6B). The disruption of deltoid crest formation can be detected by HE staining at P0, when the deltoid crest primordia are initiated (Fig. 6C). The mesenchymal cells within the presumptive DC domain of the *Col1-Pknox2* transgenic mice were accumulated at early stage as indicated by HE staining (Figure 6C). However, the later differentiation of DC cells was altered. IHC examinations using Col1 and MyoD antibodies revealed that the cell population within the deltoid crest of transgenic mice consisted of elongated muscle cells (arrows in Fig. 6D), rather than tendon or osteoblast cells. These results indicate that overexpression of *Pknox2* disrupts deltoid crest formation at the stage of osteoblast differentiation.

## Discussion

The members of the TALE family of transcriptional factors have pivotal roles in regulating limb patterning and morphogenesis as cofactors for Hox proteins. Previous studies have documented that the *Meis1* gene is a good marker for proximal limb mesenchyme and regulates limb stylopod development. The *Pbx1/2* loss-of-function mutation affects the stylopod and autopod in the hindlimb [9]. In this study, we analyzed *Pknox2* expression profiles and the effect of *Pknox2* overexpression in limb development. Misexpression of *Pknox2* in the limb mesenchyme or osteochondroprogenitor cells displayed deformities in zeugopod elements, partially resulting from perturbations of *Hox10/11* gene expression domains and decreased Bmp signaling activity in the zeugopod region. Our results suggest that *Pknox2* not only has a zeugopod-associated expression pattern, but also might be involved in the regulation of limb zeugopod and deltoid crest development. The further analysis of *Pknox2* knockout mice would add our understanding of the function of *Pknox2* in limb development.

In our study, misexpression of *Pknox2* in the limb bud mesenchyme selectively interrupted zeugopod development. We generated transgenic mice by using three different promoters. *Prx1* gene promoter drives gene expression in early mensenchymal progenitor cells including the offspring osteoblasts and chondrocytes [24]. *Col2* and *Col1* promoters activate gene expression in chondrocytes and osteoblasts, respectively. The transgenic mice driven by *Col2* and *Col1* promoter resembled partial defects of that in the *Prx1-Pknox2* mice. The timing and types of alterations in *Pknox2* transgenic mice are similar to those observed in several *Hox* gene loss-of-function mutants. The perturbation of deltoid crest development, ectopic cartilage formation in the upper elbow joint and presence of rib-like cartilage in L1 vertebrae in *Col2-Pknox2* or *Col1-Pknox2* mice are also observed in *Hoxa9/d9* double deficient mice [2]. However, no obvious perturbations of *Hox9* expression were detected in the forelimbs of the *Prx1-Pknox2* transgenic mice. On the other hand, *Hox10* functions in regulating hindlimb more than forelimb development, based on the defects in the hindlimb of the *Hoxa10/d10*-deficient mutants [26] and the triple *Hox10aaccdd* mutants [5]. Moreover, expression of *Pknox2* together with Pbx1 or Hoxa10 in 293T cells did not affect Hoxa10-mediated activation of the *Osteocalcin* promoter based on a luciferase assay (data not shown). Therefore, it is likely that perturbations in *Hoxa10/d10* expression profiles could not fully account for the forelimb zeugopod deformities caused by misexpression of *Pknox2*. Double mutants of *Hoxa11/Hoxd11* exhibit deformities in the forelimb [4], [6], similarly to the *Prx1-Pknox2* transgenic mice. Several lines of evidence have demonstrated that Pknox2 could form a heterotrimeric complex with the Hox and PBX proteins *in vitro* [14], [27]. Therefore, we speculate that *Pknox2* forms complex with Hox11 and Pbx1/2 in controlling Hox gene expression. Ectopic expression of *Meis1* in the distal limb bud also results in abnormalities in forelimb zeugopod formation [11], but disrupts radius development in a *Pbx1*-independent manner [12]. Taken together, at least some of the defects observed in *Prx1-Pknox2* mice may result from the combination of *Hox10/11* gene activity in zeugopod elements.

The defects in the *Prx1-Pknox2* mice were recapitulated to a lesser extent in mice ectopically expressing *Pknox2* in osteochondroprogenitor cells. On the other hand, early limb patterning was largely intact in both the *Prx1-Pknox2* and *Col2-Pknox2* mice, which had relatively normal skeletal segmentation in the proximal-distal axis as well as normal anterior-posterior polarity in the autopod skeletal elements. Only chondrogenic differentiation and ossification were strikingly perturbed in the zeugopod elements of transgenic mice at E12.5. Misexpression of *Pknox2* in the limb bud mesenchyme altered the expression of *Hoxa10/d10* and *Hoxa11/d11* in the zeugopod at E11.5. In fact, 5′ *Hoxd* (*Hoxd 9-13*) genes have been shown to function at the mesenchymal condensation stage of skeletal formation [28]. For instance, *Hox11* functions from the condensation stage onwards and programs chondrogenic and endochondral ossification [29]. Therefore, the anterior shift of the *Hoxa10/d10* and *Hoxd11* domains and a diminished *Hoxa11*-expression domain in the posterior side might lead to fewer mesenchymal progenitor cells available for ulna condensation and differentiation. These findings indicate that overexpression of *Pknox2* perturbs zeugopod development at the condensation and chondrogenic differentiation stages as a co-factor of Hox proteins.

Another molecular event in mice with *Pknox2* misexpression was the decrease of Bmp/Smad signaling in the zeugopod elements. Loss of *Bmp7* and *Bmpr1b* in the mouse limb leads to malformations in the ulna, radius and the deltoid crest, similar to the phenotype observed in *Prx1-Pknox2* transgenic mice [25]. Depletion of *TGFb2* also results in a slightly shortened zeugopod lacking a deltoid crest and olecranon [30]. Defects in deltoid crest formation in the *Prx1-Pknox2* mice could be recapitulated in the *Col1-Pknox2* mice but not in the *Col2-Pknox2* mice, indicating that *Pknox2* misexpression affects deltoid crest formation at the osteoblast differentiation stage. In fact, the deltoid crest is induced by the Bmp4 signal from neighboring tendon cells at E13.5 and then develops through endochondral ossification [31]. The decrease of Bmp/Smad signaling might be the consequence of altered Hox protein activity upon *Pknox2* overexpression. For instance, it has been reported that Hoxa13 directly binds to the enhancer elements of *Bmp2* and *Bmp7* to regulate their expression [32]. In addition, a variety of Hox proteins differentially suppress the transcriptional activation of Smad proteins directly via protein interaction. Protein interaction of Hoxa13-Smad5 [33], Hoxc8-Smad1 [34], Hoxd13-Smad1 [33] and Hoxa9-Smad4 [35] have been reported in various limb tissues. In addition, a trimeric complex of Smad4-Pbx1-Pknox1 has been reported to regulate the promoter of the FSHβ gene [36]. Therefore, *Pknox2* misexpression may suppress Bmp/Smad signaling activity during zeugopod development and deltoid crest formation as a co-factor for *Hox* genes.

## Supporting Information

Figure S1 **qRT-PCR analysis of** ***Hox10-11*** **gene expression in the limbs from the** ***Prx1-Pknox2*** **embryos.** qRT-PCR is performed for *Hoxa10*, *Hoxa11*, *Hoxd10* and *Hoxd11* genes in the limbs from *Prx1-Pknox2* embryos at E12.5. No obvious alteration is detected in the expression levels of these genes.
(TIF)
