# Supplementary figures and images for: Misexpression of Pknox2 in Mouse Limb Bud Mesenchyme Perturbs Zeugopod Development and Deltoid Crest Formation
Source: PLoS One. 2013 May 22;8(5):e64237. doi: 10.1371/journal.pone.0064237 (PMC3661445; doi:10.1371/journal.pone.0064237)

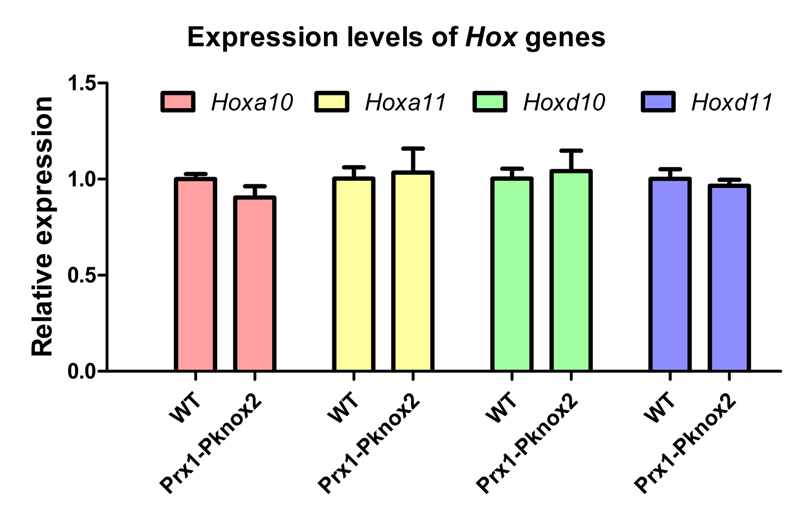

Supplement: Figure S1 — qRT-PCR analysis of Hox10-11 gene expression in the limbs from the Prx1-Pknox2 embryos. qRT-PCR is performed for Hoxa10, Hoxa11, Hoxd10 and Hoxd11 genes in the limbs from Prx1-Pknox2 embryos at E12.5. No obvious alteration is detected in the expression levels of these genes. (TIF) [file pone.0064237.s001.tif]
